# A Review of Statistical Methods for Identifying Trait-Relevant Tissues and Cell Types

**DOI:** 10.3389/fgene.2020.587887

**Published:** 2021-01-22

**Authors:** Huanhuan Zhu, Lulu Shang, Xiang Zhou

**Affiliations:** ^1^Department of Biostatistics, University of Michigan, Ann Arbor, MI, United States; ^2^Center for Statistical Genetics, University of Michigan, Ann Arbor, MI, United States

**Keywords:** trait-tissue relevance, epigenetic information, transcriptomic information, genetically regulated gene expression, gene co-expression network, eQTL information

## Abstract

Genome-wide association studies (GWASs) have identified and replicated many genetic variants that are associated with diseases and disease-related complex traits. However, the biological mechanisms underlying these identified associations remain largely elusive. Exploring the biological mechanisms underlying these associations requires identifying trait-relevant tissues and cell types, as genetic variants likely influence complex traits in a tissue- and cell type-specific manner. Recently, several statistical methods have been developed to integrate genomic data with GWASs for identifying trait-relevant tissues and cell types. These methods often rely on different genomic information and use different statistical models for trait-tissue relevance inference. Here, we present a comprehensive technical review to summarize ten existing methods for trait-tissue relevance inference. These methods make use of different genomic information that include functional annotation information, expression quantitative trait loci information, genetically regulated gene expression information, as well as gene co-expression network information. These methods also use different statistical models that range from linear mixed models to covariance network models. We hope that this review can serve as a useful reference both for methodologists who develop methods and for applied analysts who apply these methods for identifying trait relevant tissues and cell types.

## Introduction

Over the last one and half decades, genome-wide association studies (GWASs) have successfully identified and replicated many trait-relevant genetic variants in terms of single nucleotide polymorphisms (SNPs). However, most of these identified genetic variants reside outside protein-coding regions, making it challenging to understand the biological mechanism underlying these identified associations (Welter et al., 2014). Characterizing the biological mechanism underlying SNP associations is further complicated by the fact that the genetic effects of SNPs on complex traits are likely acted through a tissue-specific fashion. For example, many psychiatric disorders, such as bipolar disorder and schizophrenia, are consequences of dysfunctions of various genes, pathways, and regulatory elements in neuronal and glia cells, resulting from brain-specific genetic effects of polymorphisms (Lang et al., 2007; Uhlhaas and Singer, 2010; Fornito et al., 2015; Grunze, 2015; Xiao et al., 2017). Therefore, characterizing the function of variants in various brain tissues can help elucidate etiology of psychiatric disorders. However, for most complex traits, their trait-relevant tissues and cell types are often unknown or uncertain. As a result, identifying trait-relevant tissues and cell types and characterizing the functions of genetic variants within the relevant tissues and cell types hold the key for better understanding of disease etiology and the genetic basis of phenotypic variation (Trynka et al., 2013, 2015; Kichaev et al., 2014; Pickrell, 2014; Farh et al., 2015; Finucane et al., 2015; Li and Kellis, 2016).

Many genomic studies have been carried out in parallel to GWASs to characterize the genetic and epigenetic landscape of the human genome. These genomic studies often collect samples from multiple different tissues or cell types and characterize genomic information in a tissue- or cell type-specific fashion. For example, the ENCODE (The ENCODE Project Consortium, 2012) and Roadmap (Kundaje et al., 2015) collect various epigenetic annotation measurements in the form of open chromatin accessibility, DNase I hypersensitive sites (DHSs), and histone modifications (e.g., H3K27me3 and H3K36me3) on 16 cell lines and 111 tissues. The epigenetic information measured from these projects allows for a functional characterization of the human genome. As another example, the GTEx project collects gene expression and genotype measurements from 54 human tissues on nearly 1,000 individuals using whole-genome sequencing, whole-exome sequencing, and bulk RNA sequencing (RNA-seq) (GTEx Consortium, 2015). By paring gene expression information with genotype information, GTEx allows for the study of tissue-specific gene expression and its genetic basis in the form of expression quantitative trait loci (eQTLs) mapping. Similarly, the CommonMind project collects gene expression, open chromatin accessibility and genotype information in the dorsolateral prefrontal cortex from up to 452 patients with schizophrenia and bipolar disorder as well as healthy controls (Fromer et al., 2016). Characterizing the cortex-specific transcriptomic and epigenetic profile in CommonMind can facilitate the investigation of the molecular mechanism underlying neuropsychiatric diseases. In addition, various single cell RNA-seq (scRNA-seq) studies are being performed to collect cell type-specific gene expression measurements on tens of thousands of cells from various tissues and organs (Bacher and Kendziorski, 2016). Such cell type-specific expression profiles can be used to understand how specific cell types may underlie complex traits (Watanabe et al., 2019). Finally, existing bulk and single cell gene expression studies also facilitate the characterization of gene co-expression pattern in a tissue- or cell type-specific fashion (GTEx Consortium, 2015; Bacher and Kendziorski, 2016; Shang et al., 2020b). Tissue- or cell type-specific gene co-expression provides invaluable information on the tissue or cell type basis of disease etiology (Shang et al., 2020b). Overall, various genomic studies have provided tissue- or cell type-specific information for inferring trait-relevant tissues and cell types.

With the increasing availability of different tissue- and cell type-specific genomic datasets, many statistical methods have been recently developed to integrate these genomic data with GWASs for identifying trait-relevant tissues and cell types. These various integrative methods differ in terms of the underlying statistical model and the particular genomic information they make use of. For example, the sLDSC (stratified LD score regression) converts tissue-specific epigenetic measurements into tissue-specific SNP functional annotations and estimates to what extent different tissue-specific functional annotations explain trait heritability (Finucane et al., 2015). The inferred SNP heritability due to tissue-specific annotation is treated as a quantitative measurement for trait-tissue relevance. sLDSC is a special case of MQS (minimal norm quadratic unbiased estimation for summary statistics) and effectively relies on a method of moments (MoM) to estimate SNP heritability based on linear mixed models (Zhou, 2017). While sLDSC and MQS were initially proposed to examine one SNP annotation at a time in the presence of multiple epigenetic annotations, SMART (scalable multiple annotation integration for trait-relevant tissue identification) (Hao et al., 2018) extends these methods to simultaneously incorporate multiple tissue-specific binary and/or continuous functional annotations to facilitate consistent trait-tissue inference (Liang and Zeger, 1986; Chen et al., 2004). SMART uses the generalized estimating equation (GEE) algorithm on the same linear mixed model to achieve such inference goal. Different from using epigenetic measurements, the LDSC-SEG (sLDSC applied to specifically expressed genes) uses tissue-specific transcriptomic annotations, allowing for the inference of trait-tissue relevance with transcriptomic data (Finucane et al., 2018). Similarly, RolyPoly (a regression-based polygenic model) relies on a similar linear mixed model as used in sLDSC/MQS/SMART and creates cell type-specific annotations based on scRNA-seq data (Calderon et al., 2017). In contrast, while using the tissue-specific bulk RNA-seq expression information, the deTS method (method of decoding tissue specificity) directly examines whether the tissue-specifically expressed genes tend to be trait-associated genes using standard enrichment analysis such as the Fisher's exact test to serve as evidence of trait-tissue relevance (Pei et al., 2019). Some methods can make use of the expression quantitative trait loci (eQTLs) information in detecting trait-relevant tissues and cell types. For example, NTCS (normalized tissue causality score) uses eQTLs to assess the genetic causality behind GWASs (Ongen et al., 2017) and eQTLEnrich tests whether eQTLs from a given tissue and/or cell type are significantly enriched for trait associations (Gamazon et al., 2018). Alternatively, other methods measure the trait-tissue relevance by evaluating the proportion of phenotypic variance explained by genetically regulated expression levels (GReX) in different tissues. For example, IGREX (impact of genetically regulated expression) (Cai et al., 2020) and RhoGE (Mancuso et al., 2017) obtain the predicted GReX in tissues and use the association evidence of tissue-specific GReX with the trait for inferring trait-relevant tissues. Finally, CoCoNet (composite likelihood-based covariance regression network model) (Shang et al., 2020b) integrates GWAS data with tissue- or cell type-specific gene co-expression patterns obtained from bulk or single cell gene expression studies based on a network model. In particular, CoCoNet expresses gene-level effect sizes for the given GWAS trait as a function of the tissue-/cell type-specific adjacency matrix and infers how a tissue is relevant to the given trait by examining how effective the tissue-specific gene co-expression network is for predicting gene-level association pattern with the trait.

**Figure 1:**
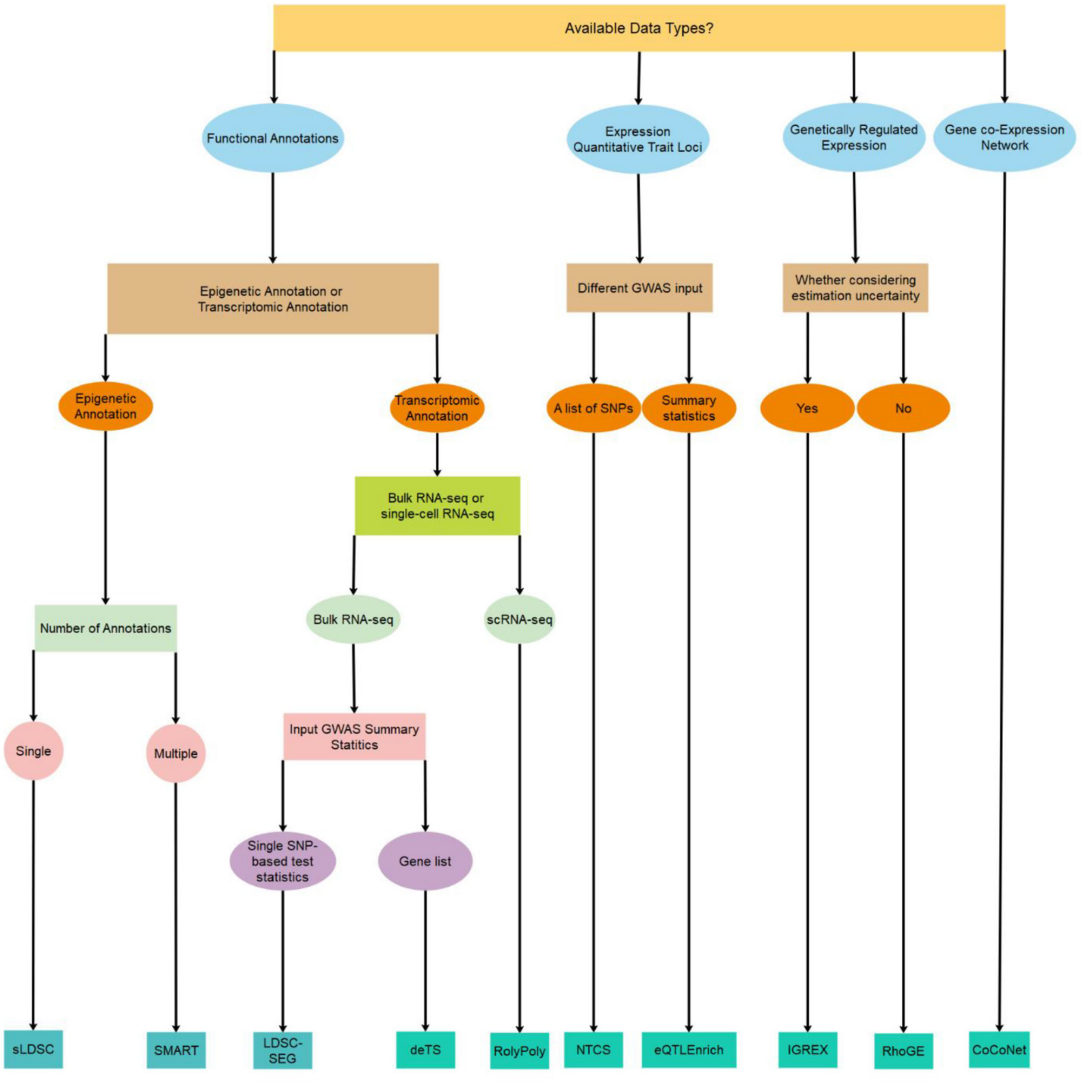
A decision tree on which method to use for identifying trait-relevant tissues/cell types based on the availability of data types.

**Figure 2:**
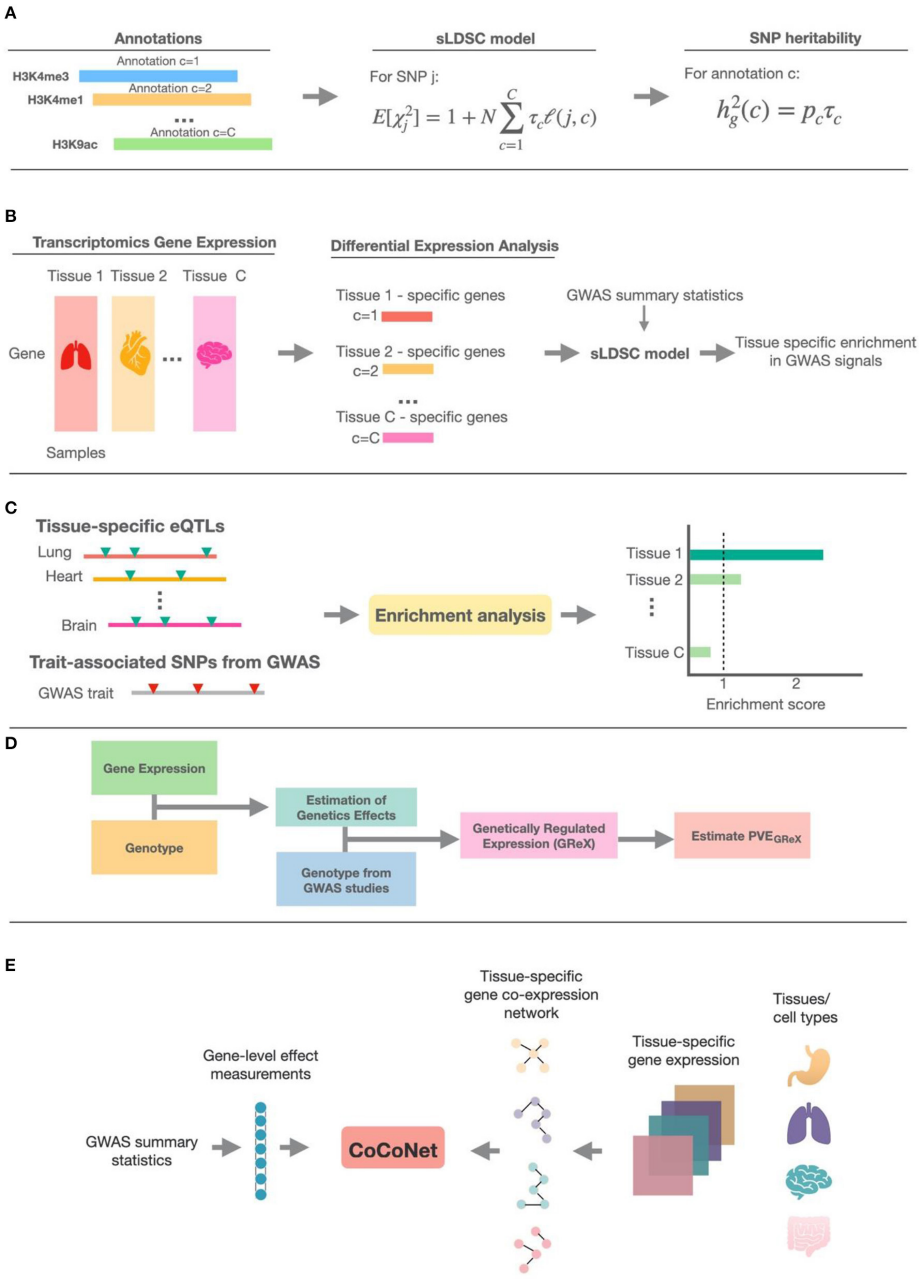
The schematic illustration of methods in the five different categories. **(A)** The general schema of methods that make use of epigenetic annotation information; sLDSC is shown as the detailed example. **(B)** The general schema of methods that use tissue-specific transcriptomic annotation information; these methods first define specifically expressed genes (SEGs) based on differential expression analysis, then construct genomic annotations from the SEGs, and finally use sLDSC to perform trait-tissue relevance inference. **(C)** The schema of methods that test for enrichment of trait associations among eQTLs in each tissue. **(D)** The general schema of methods that obtain the estimated genetically regulated expression (GReX) and use the proportion of phenotypic variance explained by GReX (PVE_GReX_) to measure the trait-tissue relevance. **(E)** The schema of methods that make use of tissue-specific gene co-expression networks; CoCoNet is shown as the detailed example.

Despite the abundance of integrative methods developed for trait-tissue relevance inference, however, a comprehensive review is currently lacking for summarizing the technical details and benefits of each of the above methods. Previous reviews on tissue-trait relevance inference often focus on a limited number of methods that use only functional annotations (Cano-Gamez and Trynka, 2020). To fill this critical knowledge gap, we provide a systemic review on ten different integrative methods for trait-tissue relevance inference. These methods are organized into four main categories based on the tissue- or cell type-specific genomic information they reply on. For each method in turn, we describe the input genomic data types, the detailed statistical model and computational algorithm, the output for evaluating trait-tissue relevance, and the main results obtained in the original study. A summary of these methods is provided in Table 1 and Figure 1, with a brief schematic illustration of each type of methods provided in Figure 2. We hope that this review can serve as a useful reference for practitioners who are interested in identifying the causal tissues/cell types of GWAS traits and understanding the SNP association with complex traits in a tissue-specific fashion, as well as for methodologists who develop computational methods for quantifying trait-tissue relevance.

**Table 1:** A summary of statistical methods for trait-tissue relevance inference.

**Genomic information**	**Method**	**GWAS inputs**	**Measurements**	**Strengths**	**Limitations**	**References**
Epigenetic annotations	sLDSC	SNP-based Summary statistics	*p-*values	It extends the commonly used LDSC approach by partitioning SNPs into different functional categories and determining the contribution of each category to trait heritability; can test one annotation while controlling for other annotations in the model.	Examines one annotation at a time; relies on the standard linear mixed model that assumes a polygenic genetic architecture; uses method of moments for model fitting.	Finucane et al., 2015
	SMART	Either individual-level phenotype and genotype data or summary statistics	Posterior probabilities	It handles multiple binary and/or continuous annotations simultaneously; uses the computationally efficient GEE method to estimate and make inference on annotation coefficients.	Relies on the standard linear mixed model that assumes a polygenic genetic architecture.	Hao et al., 2018
Transcriptomic annotations	LDSC-SEG	SNP-based summary statistics	*p-*values	Same as the sLDSC model; effectively creates a gene level annotation by annotating SNPs in genes that are specifically expressed in a tissue to one and annotating the remaining SNPs to zero.	Model performance highly depends on the gene expression data, which is used to determine tissue specificity of gene expression and subsequently tissue specific SNP annotations; sensitive to gene expression correlation across cell and tissue types.	Finucane et al., 2018
	RolyPoly	SNP-based summary statistics	*p-*values	Similar to the sLDSC model; integrates scRNA-seq data with GWAS; jointly analyzes gene expression from multiple tissues or cell types; prioritizes trait-relevant cell types and genes.	Model performance highly depends on the gene expression data used; sensitive to gene expression correlation across cell and tissue types.	Calderon et al., 2017
	deTS	A list of trait-associated genes	*p-*values	Applicable when only a list of GWAS significant genes are available.	Model performance highly depends on the gene expression data; there is not a commonly accepted threshold for defining trait-associated genes, and different thresholds may result in different sets of genes and thus different enrichment results.	Pei et al., 2019
eQTL information	NTCS	A list of trait-associated and null SNPs	Ranking of tissues based on adjusted fold-enrichment	Rank genes in terms of their contribution to trait-tissue relevance.	No publicly available tools; model implementation is redundant and difficult to replicate.	Ongen et al., 2017
	eQTLEnrich	GWAS summary statistics	*p-*values	Both tissue-shared and tissue-specific regulatory effects of eQTLs are analyzed.	The adjusted fold-enrichment used for ranking tissues in eQTLEnrich is correlated with GWAS sample size.	Gamazon et al., 2018
Genetically regulated expression (GReX)	IGREX	Either individual-level phenotype and genotype data or summary statistics	*p-*values	Measures the phenotypic variance explained by GReX; can analyze both GWAS individual-level and summary data.	Uses REML for inference, which can be time consuming.	Cai et al., 2020
	RhoGE	SNP-based summary statistics	*p-*values	Measures the phenotypic variance explained by GReX.	Uses a two-stage regression for inference, which may fail to account for estimation uncertainty in the first stage.	Mancuso et al., 2017
Gene co-expression network	CoCoNet	Either individual-level phenotype and genotype data or summary statistics	Ranking of tissues based on log-likelihood	Incorporates tissue-specific gene co-expression networks constructed from either bulk or single cell RNA sequencing (RNAseq) studies with GWAS data; is scalable to tens of thousands of genes.	Currently only focuses on ranking tissues for a given disease.	Shang et al., 2020b

*These methods make use of different genomic information (1st column), GWAS inputs (3rd column), and different measurements (4th column) for trait-tissue relevance inference, strengths (5th column), and limitations (6th column) of each method*.

## Methods Based on Tissue-Specific SNP Functional Annotations

Here, we describe the first category of methods for trait-tissue relevance inference. The first category of methods makes use of SNP functional annotations. Exemplary methods include sLDSC (Finucane et al., 2015) and SMART (Hao et al., 2018) that make use of epigenetic annotations; and LDSC-SEG (Finucane et al., 2018), deTS (Pei et al., 2019), and RolyPoly (Calderon et al., 2017) that make use of transcriptomic annotations. The key idea behind these methods is to estimate the contribution of tissue-/cell type-specific functional annotations to SNP heritability for the GWAS trait of interest.

### Methods That Use Epigenetic Annotations

In parallel to trait mapping efforts, large-scale functional genomic studies have yielded a rich source of epigenetic annotations (The ENCODE Project Consortium, 2012; Akbarian et al., 2015; Kundaje et al., 2015; Stunnenberg et al., 2016). Various discrete and continuous epigenetic annotations are being developed to describe and characterize the biological function of genetic variants (Kellis et al., 2014; Carithers and Moore, 2015; Dixon et al., 2015). For example, we can now classify genetic variants based on their biochemical function as measured by histone modification, DNase I hypersensitive sites (DHSs), metabolomic QTL evidence, and/or a combination of all these measurements in the form of chromatin states (Pique-Regi et al., 2011; Ernst and Kellis, 2012; McVicker et al., 2013). Often times, these epigenetic annotations are tissue specific and/or cell type specific, allowing characterizing SNP functions in a tissue- or cell type-specific fashion. Paring such tissue-specific SNP epigenetic annotations with SNP association evidence with the GWAS trait allows us to infer trait-tissue relevance. Here, we introduce two methods, sLDSC and SMART, that make use of epigenetic information for trait-tissue relevance inference. In the present review, we simply refer to each tissue-specific epigenetic annotation (e.g., H3K4me1, H3K4me3, and H3K9ac) as a functional category.

#### sLDSC

The sLDSC (Finucane et al., 2015) estimates how a tissue-/cell type-specific functional annotation contributes to the SNP heritability of the GWAS trait as evidence for trait-tissue relevance inference. Specifically, for each examined tissue in turn, sLDSC first partitions SNPs into *C* different non-overlapping functional categories based on tissue-specific epigenetic annotations. We use *H*_*c*_ (*c* = 1, …, *C*) to denote the set of SNPs that belong to the *c*-th category. For example, *C* could be three, with *H*_1_ = H3K4me1 that consists of SNPs that are inside or nearby H3K4me1 peaks in the examined tissue, *H*_2_ = H3K4me3 that consists of SNPs that are inside or nearby H3K4me3 peaks, and *H*_3_ = H3K9ac that consists of SNPs that are inside or nearby H3K9ac peaks. We denote $\chi_{j}^{2}$ as the marginal chi-square statistics for the *j*-th SNP association with the trait. sLDSC considers the following model on the marginal chi-square statistic:

(1) $$\begin{array}{l} E\left[\chi_{j}^{2}\right]=1+N\overset{C}{\underset{c=1}{\sum }}\tau_{c}\ell \left(j,c\right), \end{array}$$

where $\ell \left(j,c\right)=\underset{j^{\prime }\in H_{c}}{\sum }r_{jj^{\prime }}^{2}$ is the LD score of the *j*-th SNP with respect to category *c*, with $r_{jj^{\prime }}^{2}$ being the R-squared value between *j*-th SNP and *j*′-th SNP that is in the set *H*_*c*_; and τ_*c*_ represents the per-SNP heritability of category *H*_*c*_. The total SNP heritability explained by the examined functional annotation *H*_*c*_ is defined as $h_{g}^{2}\left(c\right)=p_{c}\tau_{c}$ with *p*_*c*_ being the number of SNPs in category *c*. By replacing $E\left[\chi_{j}^{2}\right]$ with the observed GWAS marginal association statistic $\chi_{j}^{2}$ and solve Equation (1), sLDSC can obtain the estimate of τ_*c*_, ${\hat{\tau }}_{c}$, and subsequently ${\hat{h}}_{g}^{2}\left(c\right)$. With the standard error of ${\hat{h}}_{g}^{2}\left(c\right)$ estimated using a jackknife procedure (Quenouille, 1956), sLDSC can further compute a z-score ${\hat{h}}_{g}^{2}\left(c\right)/se\left({\hat{h}}_{g}^{2}\left(c\right)\right)$ and a subsequent *p-*value as a measurement of the tissue/cell type relevance to the GWAS trait based on the functional annotation *c*. In the original paper, the sLDSC method is applied to analyze 17 complex diseases and traits using one functional annotation at a time. By analyzing cell type-specific functional annotations, sLDSC identified many cell type relevance to traits. Examples include the relevance of central nervous system cell types to body mass index, age at menarche, year of education, and smoking status.

#### SMART

sLDSC examines one functional annotation at a time. However, analyzing one epigenetic annotation at a time fails to incorporate the rich information contained in various other annotations that likely characterize other functionality of variants (Lu et al., 2016, 2017; He et al., 2017). For example, some annotations are designed to evaluate the function of a variant in determining the protein structure, while some other annotations are designed to quantify its ability to regulate gene expression. Even categories that belong to the same epigenetic annotation may characterize substantially different functions of a variant. For example, H3K4me1 is used to annotate enhancers while H3K4me3 is used to annotate promoters. Therefore, it is desirable to make use of multiple epigenetic annotations to obtain consistent and robust trait-tissue relevance inference results. A key step that facilitates the incorporation of multiple epigenetic annotations is the discovery that the data generating model underlying sLDSC is a standard linear mixed model and that sLDSC fits the linear mixed model using the method of moments (MoM) (Zhou, 2017). Indeed, sLDSC is practically a special case of MQS, which provides a unified framework for variance component estimation in linear mixed models (Zhou, 2017). Building upon the same linear mixed model that sLDSC and MQS use, SMART (Hao et al., 2018) was developed to incorporate multiple tissue-/cell type-specific epigenetic annotations for trait tissue/cell type inference. In particular, SMART allows for the incorporation of multiple tissue-specific binary and continuous epigenetic annotations. For example, a tissue-specific binary histone annotation can be an indicator that indicates whether the SNP resides inside the peak regions of the histone mark, while a tissue-specific continuous histone annotation can be an average of counts in the histone peak region. Importantly, because of its reliance on a data generative linear mixed model, SMART can be applied to handle either individual-level GWAS data or summary statistics. For individual-level GWAS data, SMART models the phenotype as

(2) $$\begin{array}{l} \begin{array}{c} \text{y}=\tilde{\text{G}}\gamma +\varepsilon_{\text{y}}, \end{array} \end{array}$$

where **y** is a vector of phenotypes for *N* GWAS samples; $\tilde{\text{G}}$ is an *N* × *p* genotype matrix measured from the same *N* samples and *p* genome-wide SNPs; ***γ*** is a *p*-vector of effect sizes; and $\varepsilon_{y}\sim N\left(0_{N},\;\sigma_{y}^{2}{\text{I}}_{N}\right)$ is the *N*-vector error term, where **0**_*N*_ represents an *N*-vector of zeros and **I**_*N*_ represents an *N*-dimensional identity matrix. The phenotype **y** and each column of the genotype matrix $\tilde{\text{G}}$ are standardized to have zero mean and unit standard deviation, allowing us to ignore the intercept in Equation (2). SMART assumes that all SNPs are characterized by a set of *s* functional annotations. For the *j*-th SNP, we use a (*s* + 1)-vector $F_{j}={\left(1,\;F_{j1},\;\ldots ,F_{js}\right)}^{T}$ to denote its annotation values across *s* functional epigenetic annotations, where the first value 1 corresponds to the intercept. Here, each of *F*_*j*1_, …, *F*_*js*_ can either be a binary value or a continuous value. With the SNP annotations, SMART assumes that the SNP effect size γ_*j*_ follows a normal distribution with zero mean and SNP-specific variance that is a function of the annotation vector,

(3) $$\begin{array}{l} \begin{array}{c} \gamma_{j}\sim N\left(0,\frac{\sigma_{j}^{2}}{p}\right),\;\sigma_{j}^{2}=F_{j}\alpha^{*}, \end{array} \end{array}$$

where $\alpha^{*}=\left(\begin{array}{c} \alpha_{0}\\ \alpha \end{array}\right)$ is a (*s* + 1)-vector of coefficients that include an intercept α_0_ and a *s*-vector of annotation coefficients ***α***. To evaluate the joint contribution of multiple annotations to genetic effect sizes, SMART performs parameter inference using the generalized estimation equation (GEE) (Liang and Zeger, 1986). Use of GEE not only enables scalable computation, but also allows for the use of GWAS summary statistics based on the same model characterized by Equations (2) and (3). By applying GEE, SMART obtains point estimates $\hat{\alpha }$ and their covariance matrix $Var\left(\hat{\alpha }\right)$, which allow for the computation of the multivariate Wald statistic, ${\hat{\alpha }}^{T}Var{\left(\hat{\alpha }\right)}^{-1}\hat{\alpha }$. The Wald statistic is further modeled as a mixture of two non-central chi-squared distributions for classifying tissues into trait-relevant and trait-irrelevant groups. An expectation-maximum (EM) algorithm is then applied to the chi-squared mixture to infer the posterior probability of a tissue being a trait-relevant tissue.

In the original paper, SMART analyzed 43 traits from 29 GWAS studies and obtained many trait-relevant tissues and cell types. For example, SMART identified the central nervous system (CNS) tissues to be the most trait-relevant for psychiatric disorders (e.g., schizophonia, Alzheimer's disease) and neurological related traits (e.g., years of education, childhood BMI). These results are consistent with existing literature. For example, searching the trait-tissue pair schizophrenia-CNS on PubMed yielded 17,720 hits while searching for the trait-tissue pair Alzheimer-CNS yielded 34,395 hits, supporting their clear relevance. As another example, SMART identified the bone and connective tissues to be related to height and femur neck bone mineral density, and the blood/immune tissues to be related to immune diseases (e.g., Rheumatoid Arthritis, type 1 diabetes). These results are also in line with literature: PubMed search for height-BoneConnective yielded 13,644 hits and search for RA-BloodImmune yielded 6,868 hits, supporting their relevance.

### Methods That Use Transcriptomic Annotations

Besides epigenomic studies, many gene expression studies have been carried out to characterize the transcriptomic landscape of various tissues and cell types (The ENCODE Project Consortium, 2012; GTEx Consortium, 2015; Kundaje et al., 2015). These tissue- and cell type-specific gene expression information can be invaluable for inferring trait-tissue relevance (Hu et al., 2011; Slowikowski et al., 2014; Pers et al., 2015; Gormley et al., 2016). In this section, we introduce three methods that make use of gene expression data in the form of transcriptomic annotations. These methods include LDSC-SEG (Finucane et al., 2018) and deTS (Pei et al., 2019) that make use of bulk RNA-seq expression data, and RolyPoly (Calderon et al., 2017) that makes use of single-cell RNA-seq expression data.

#### LDSC-SEG

LDSC-SEG consists of two separate steps. The first step of LDSC-SEG is a differential expression analysis on the gene expression data to identify a set of genes that are specifically expressed in certain tissues. These tissue specific genes are referred to either as specifically/differentially expressed genes (SEGs) or tissue-specific genes (TSGs). In the differential expression analysis, LDSC-SEG examines one gene at a time. For the given gene, LDSC-SEG contrasts the gene expression level of samples collected in a focal tissue (e.g., brain-cortex) with those of samples collected in all other tissues that are not in the same tissue category as the focal tissue (i.e., non-brain tissues). Because tissues within each tissue category tend to share similarly expressed genes, excluding the tissues in the same tissue category in the differential expression analysis step becomes the key to ensure robust detection of SEGs. Indeed, such differential expression analysis allows for the inclusion of as many genes as possible that are highly expressed in the focal tissues but not in tissues from other tissue categories. The SEG evidence for a gene is typically characterized by a t-statistic, with a higher value indicating that the gene is more specifically/differentially expressed in the focal tissue. With the differential expression analysis results, LDSC-SEG ranks all genes in a descending order based on their t-statistics. LDSC-SEG then defines SEGs as the top 10 percentage of all genes. The identification of SEGs allows LDSC-SEG to create a binary SNP annotation in a tissue specific fashion. In particular, for each tissue at a time, LDSC-SEG annotates the SNP to be one if the SNP resides within 100 kb of the transcription start site of any SEG and annotates it to be zero otherwise. With the tissue-specific binary annotation, LDSC-SEG then performs the second step of applying the sLDSC method described in the previous section to estimate the proportion of SNP heritability explained by each tissue-specific binary SNP annotation. The resulting test statistic from sLDSC is then served as a relevance evidence between the tissue and trait.

In real data applications, LDSC-SEG analyzed GWAS summary statistics for 48 diseases and traits and found significant tissue-/cell type-specific enrichments for 34 traits. Several of these findings recapitulate known biology. For example, immunological traits exhibit immune tissue-type enrichments; psychiatric traits exhibit strong brain-related tissue enrichments; and type II diabetes exhibits enrichments in the pancreas. LDSC-SEG also validated several recent genetic analyses results, including robust brain-specific enrichments for smoking status, years of education, body mass index, and age at menarche.

#### deTS

deTS also consists of two-steps. The first step of deTS also consists of a differential expression analysis as in the first step of LDSC-SEG. The only minor difference there is the definition of SEGs: while LDSC-SEG defines top 10% as SEGs, deTS defines top 5% as SEGs. However, the second step of deTS relies on an enrichment analysis rather than sLDSC. Specifically, deTS implements Fisher's exact approach to test whether the SEGs are enriched in the focal tissue or not. The Fisher's exact test builds upon a two-by-two contingency table, where the two rows represent the number of SEGs vs. the number of non-SEGs in the tissue, while the two columns represent the number of trait-associated genes vs. the number of non-trait-associated genes. Here, the trait-associated gene is defined based on a gene-level *p-*value threshold of 5 × 10^−3^, where the *p-*value is calculated from a gene-based test (Lamparter et al., 2016). In the original study, deTS is applied to analyze GWAS summary statistics for 26 traits. deTS found that artery tissues were primarily associated with anthropometric trait, liver was primarily associated with metabolic traits, blood and spleen were primarily associated with immune-related traits, and brain tissues were primarily associated with neurodegenerative/neuropsychiatric diseases.

#### RolyPoly

RolyPoly (Calderon et al., 2017) is specifically developed for single cell expression studies. It consists of the same two steps as LDSC-SEG. In the first step, RolyPoly uses a slightly different approach than LDSC-SEG to define the SEGs. Specifically, for each tissue, RolyPoly ranks all genes in a descending order based on the normalized expression values and define the top 20% of genes as SEGs. Afterwards, RolyPoly creates a binary SNP annotation based on whether a SNP resides within a 10 kb window nearby the transcription start site of any SEGs. In the second step, RolyPoly applies the same linear mixed model as used in sLDSC for inference (Finucane et al., 2015). In real data analysis, RolyPoly identified significant relevance of oligodendrocytes and fetal replicating cells with schizophrenia.

## Methods Based on Expression Quantitative Trait Loci Information

In recent years, expression mapping studies have succeeded in identifying many cis-acting genetic variants known as cis-eQTLs that are associated with gene expression levels (Schadt et al., 2003; Morley et al., 2004; Lappalainen et al., 2013; Battle et al., 2014). The identified eQTLs can help elucidate the molecular mechanisms underlying human disease associations and facilitate the identification of biological pathways underlying disease etiology. For example, it has been shown that the GWAS variants frequently colocalize and likely share functional effects with eQTLs (Nica et al., 2010; Nicolae et al., 2010; Grundberg et al., 2012; Shang et al., 2020a). Thus, at least some of these variants influence traits through regulatory effects. In addition, the identified eQTLs in multiple tissues and/or cell types can help interpret the GWAS results through linking non-coding genomic regions to gene functions and identifying causal tissues/cell types behind the genetic associations (Nica and Dermitzakis, 2008; Montgomery and Dermitzakis, 2011; Grundberg et al., 2012). In this section, we will introduce two methods, NTCS (Ongen et al., 2017) and eQTLEnrich (Gamazon et al., 2018), that make use of tissue- and cell type-specific eQTL information to infer the trait-relevant tissues and cell types that are behind genetic causality.

### NTCS

For a given tissue, NTCS makes use of a list of significant eQTLs that are not in linkage disequilibrium (LD) with each other along with their colocalized GWAS variants. These eQTLs are obtained from a conditional eQTL mapping analysis, performed through, for example, FastQTL (Welter et al., 2014). The identified eQTLs are overlapped with common variants downloaded from the NHGRI-EBI GWAS catalog (Storey and Tibshirani, 2003) to obtain a list of eQTLs that have GWAS significance (*P* < 5e−8). These eQTLs are denoted as real GWAS variants, GWAS variants, or GWAS-associated variants.

The NTCS method first uses the Regulatory Trait Concordance (RTC) (Nica et al., 2010) approach to detect colocalized variants between the GWAS study and the eQTL study while properly accounting for LD. The resulted RTC score is then converted to a probability value that measures the sharing between a GWAS variant and an eQTL in a tissue, or between two eQTLs in a pair of tissues based on Bayes' theorem:

(4) $$\begin{array}{l} P\left(shared|RTC=rtc\right)\\ =\frac{P\left(RTC=rtc|shared\right)\cdot \pi_{1}}{P\left(RTC=rtc|shared\right)\cdot \pi_{1}+P\left(RTC=rtc|notshared\right)\cdot \pi_{0}}, \end{array}$$

where *P*(*shared*) = π_1_ is a π_1_ statistics and π_0_ = 1 − π_1_. When calculating the probability of sharing between the GWAS variants and eQTLs in a given tissue, the π_1_ statistics is calculated from eQTL *p-*values in the tissue and GWAS variants. When calculating the probability of sharing between two eQTLs in a pair of tissues, the π_1_ statistics is calculated from eQTL *p-*values in the two tissues. Both *P*(*RTC* = *rtc*|*not shared*) and *P*(*RTC* = *rtc*|*shared*) are estimated through simulations, where the RTC scores are simulated under both the null and alternative hypotheses. Specifically, for each coldspot that has colocalized GWAS and eQTL variants (eQTL_real_), under the null hypothesis (H_0_) where GWAS and eQTL are tagging two different variants, two hidden causal variants (GWAScausal and eQTLcausal) are randomly selected. Under the alternative hypothesis (H_1_) where GWAS and eQTL are tagging the same variant, one hidden causal variant (eQTL_causal_) is randomly selected. In both hypotheses, the GWAS and eQTL variants are randomly selected from the variants that are in linkage disequilibrium with the hidden causal variants with *r*^2^ ≥ 0.5. Afterwards, gene expression is simulated based on the eQTL_real_ effect size. The RTC analyses are then performed under H_0_ and H_1_, each for 200 times. For each coldspot, the total 400 simulated RTC scores under H_0_ and H_1_ are merged and sorted to obtain a point probability. Finally, for each GWAS trait in each given tissue and each eQTL that colocalizes with a GWAS variant, NTSC defines a normalized GWAS variant-eQTL probability as the probability of the GWAS variant and eQTL tagging the same functional effect divided by the sum of the tissue-sharing probabilities for the eQTL in that tissue. Intuitively, tissue-specific eQTLs would more likely be a GWAS variant than tissue non-specific eQTLs that are shared across tissues. Therefore, for each GWAS trait in each given tissue, NTCS defines a normalized tissue causality score (NTCS) and a null NTCS as follows:

(5) $$\begin{array}{l} NTCS=\frac{1}{p_{2}}\times \overset{p_{1}}{\underset{j=1}{\sum }}\frac{P\left(SNP_{j}-eQTL_{j}\;shared|rtc\right)}{P\left(eQTL_{j}\;shared|rtc\right)}, \end{array}$$

(6) $$\begin{array}{l} Null\;NTCS=\frac{p_{1}}{p_{0}p_{2}}\times \overset{p_{0}}{\underset{j=1}{\sum }}\frac{P\left({null\;SNP}_{j}-eQTL_{j}\;shared|rtc\right)}{P\left(eQTL_{j}\;shared|rtc\right)}, \end{array}$$

where *p*_1_ is the number of GWAS-associated variants for the trait; *p*_2_ is the total number of eQTLs in a given tissue; *p*_0_ is the number of GWAS-null variants; *P*(*SNP*_*j*_ − *eQTL*_*j*_ *shared*|*rtc*) is the probability that a GWAS variant (i.e., *SNP*_*j*_) and *eQTL*_*j*_ tagging the same functional effect; and *P*(*eQTL*_*j*_ *shared*|*rtc*) is defined in Equation (4). An enrichment metric is further defined as $\frac{NTCS}{null-NTCS}$. The tissues with an enrichment metric greater than one are likely the causal tissues for the diseases/traits. To create a *p-*value for testing trait-relevance of each tissue, NTCS first selects a *null* GWAS variant to match each of the GWAS variant, based on minor allele frequency and distance to the closest transcription start site. Afterwards, NTCS repeats the above enrichment metric calculation using the set of null GWAS variants, examines one tissue at a time, compares the tissue metric for the disease-associated variants to the metric observed under the null for that tissue, and calculates a corresponding *p-*value based on a Mann-Whitney test that compares the distribution containing each of the j-th elements in Equation (5) and (6) for the real GWAS and under the null. In the NTCS paper, NTCS method discovers that liver is the tissue most likely to be causal in most of the GWAS traits. Brain tissues are the top tissues relating to traits like schizophrenia, height, and age of onset of puberty.

### eQTLEnrich

eQTLEnrich is a rank- and permutation-based method that aims to test for enrichment of trait associations among eQTLs in each tissue. For a given GWAS trait, for each of the tissues with eQTLs, eQTLEnrich first finds the most significant cis-eQTL per eGene, and then extracts the GWAS variant association *p-*values for each set of eQTLs. Afterwards eQTLEnrich tests for the enrichment of the distribution of GWAS *p-*values for each set of eQTLs in the corresponding tissue. The distribution of the GWAS *p-*values for each set of eQTLs is tested for enrichment of highly ranked trait associations compared to an empirical null distribution sampled from non-significant variant-gene expression associations.

Specifically, eQTLEnrich first computes the fold-enrichment for each GWAS-tissue pair. The fold-enrichment is defined as the fraction of eQTLs with GWAS variant *p* < 0.05 compared to expectation. Similarly, eQTLEnrich also computes fold-enrichment values for randomly sampled sets of non-significant variant-gene expression associations of equal size to the eQTL set, matching the distance of eQTL to TSS of the target gene, MAF, and number of proxy variants (at *r*^2^ ≥ 0.5), to account for LD. Then eQTLEnrich computes an enrichment *p-*value as the fraction of permutations with similar or higher fold-enrichment than the observed value. Finally, eQTLEnrich computes an adjusted fold-enrichment by dividing the fold-enrichment for a specific GWAS-tissue pair by the fold-enrichment of all non-significant variant-gene expression associations with GWAS *P* < 0.05 for the tissue-trait pair. The eQTLEnrich method is applied to analyze 18 complex diseases and traits on 44 GTEx tissues and identifies many trait-relevant tissues. Examples include the relevance of left heart ventricle and adipose visceral omentum to type I diabetes, ovary and artery coronary to coronary artery disease, and hippocampus to Alzheimer's disease.

## Methods Based on Tissue-Specific Genetically Regulated Expression Levels

Here, we describe the third category of methods for trait-tissue relevance inference. The third category of methods use information from genetically regulated expression levels (GReX) that are constructed in a tissue specific fashion. GReX measures the part of gene expression levels that can be predicted by (cis-)SNPs (Gamazon et al., 2015). In a given tissue, GReX is constructed for each gene by fitting a prediction model that relates the gene expression level to the cis-SNPs. Common prediction models for GReX construction include elastic net (Zou and Hastie, 2005), BSLMM (Zhou et al., 2013), and DPR (Zeng and Zhou, 2017). Constructed GReX is often tested with the GWAS trait for association evidence through transcriptome-wide association studies (TWAS) (Gamazon et al., 2015; Gusev et al., 2016). Indeed, GReX of many genes have been identified to be associated with diseases and disease-related complex traits. In this section, we will introduce two methods, IGREX (Cai et al., 2020) and RhoGE (Mancuso et al., 2017), that rely on GReX to infer trait-tissue relevance. Both methods effectively are built upon the same model but rely on different algorithms for model inference.

Specifically, both methods consider two separate models, one for the gene expression study and the other for the GWAS. In the gene expression study, both methods examine one tissue and one gene at a time. For the *m*-th gene in the tissue, both methods consider the following linear model for modeling the relationship between gene expression and genotypes of cis-SNPs,

(7) $$\begin{array}{l} \begin{array}{c} {\text{z}}_{m}={\text{G}}_{m}{\text{w}}_{m}+\varepsilon_{z}, \end{array} \end{array}$$

where **z**_*m*_ is an *n*-vector of expression values measured from a focal tissue, with *n* being number of available samples in this tissue; **G**_*m*_ is an *n* × *p* genotype matrix for the same *n* samples and *p* cis-SNPs for the given gene; **w**_*m*_ is a *p*-vector of SNP effect sizes on the gene expression; and $\varepsilon_{z}\sim N\left(0_{n},\;\sigma_{z}^{2}{\text{I}}_{n}\right)$ is the residual error term. The gene expression **z**_*m*_ and each column of genotype matrix **G** are standardized, allowing us to ignore the intercept term in Equation (7). The genetic effects on gene expression is assumed to follow a normal distribution *a priori*, with ${\text{w}}_{m}\sim N\left(0_{p},\;\sigma_{w}^{2}{\text{I}}_{p}\right)$.

In the GWAS data, both methods consider the following regression model that relates the phenotype to genotype:

(8) $$\begin{array}{l} \text{y}={\tilde{\text{G}}}_{r}\gamma +\overset{M}{\underset{m=1}{\sum }}\beta_{m}{\tilde{\text{G}}}_{m}{\text{w}}_{m}+\varepsilon_{y}, \end{array}$$

where **y** and **ε**_*y*_ are defined as in Equation (2); ${\tilde{\text{G}}}_{m}$ is the *N* × *p* genotype matrix for *p* cis-SNPs in the given gene; **w**_*m*_ is the same SNP effects on gene expression as defined in Equation (7); the scalar $\beta_{m}\sim N\left(0,\sigma_{\beta }^{2}\right)$ represents the genetic effect of GReX (i.e., ${\tilde{\text{G}}}_{m}{\text{w}}_{m}$) on **y** and can be interpreted as the causal effect of GReX on **y** (Yuan et al., 2019; Zhu and Zhou, 2020); and $\gamma \sim N\left(0,\sigma_{\gamma }^{2}{\text{I}}_{q}\right)$ is the *q*-length vector of alternative genetic effects; note that ${\tilde{\text{G}}}_{\gamma }$ is not the same genotype matrix as ${\tilde{\text{G}}}_{m}$, and the *q* SNPs in ${\tilde{\text{G}}}_{\gamma }$ are those who show direct horizontal effects on **y**, such as the trans-eQTLs and SNPs associated with alternative splicing events (Matlin et al., 2005).

Above, the proportion of phenotypic variance explained by GReX is calculated as

(9) $$\begin{array}{l} \begin{array}{c} PVE_{GReX}=\frac{Var\left(\sum_{m}\beta_{m}{\tilde{\text{G}}}_{m}{\text{w}}_{m}\right)}{Var\left(\text{y}\right)}. \end{array} \end{array}$$

### IGREX

IGREX (Cai et al., 2020) relies on a two-stage method to perform inference for the model defined in Equations (7) and (8). Specifically, IGREX first estimates the posterior distribution of genetic effects on expression based on Equation (7) and obtains the posterior distribution **w**_*m*_|**z**_*m*_, **G**_*m*_ ~ *N*(***μ***_*m*_, **Σ**_*m*_) for each gene *m*. Afterwards, IGREX treats the posterior distribution **w**_*m*_|**z**_*m*_, **G**_*m*_ from Equation (7) as the prior distribution for Equation (8), and obtain the estimates of $\sigma_{\beta }^{2}$, $\sigma_{\gamma }^{2}$ and $\sigma_{y}^{2}$ using either the method of moments (MoM) or REML. Finally, the estimate of *PVE*_*GReX*_ is obtained by

(10) $$\begin{array}{l} \begin{array}{c} {\hat{PVE}}_{GReX}=\frac{tr\left(\sum_{m}{\hat{\sigma }}_{\beta }^{2}{\tilde{\text{G}}}_{m}\left(\mu_{m}\mu_{m}^{T}+\Sigma_{m}\right){\tilde{\text{G}}}_{m}^{T}\right)}{tr\left(\sum_{m}{\hat{\sigma }}_{\beta }^{2}{\tilde{\text{G}}}_{m}\left(\mu_{m}\mu_{m}^{T}+\Sigma_{m}\right){\tilde{\text{G}}}_{m}^{T}+{\hat{\sigma }}_{\gamma }^{2}{\tilde{\text{G}}}_{r}{\tilde{\text{G}}}_{r}^{T}+{\hat{\sigma }}_{y}^{2}I_{N}\right)}. \end{array} \end{array}$$

In the above two-step estimation procedure, IGREX relies on the posterior distribution **w**_*m*_|**z**_*m*_, **G**_*m*_ to account for estimation uncertainty associated with **w**_*m*_ in Equation (8). Given the point estimate ${\hat{PVE}}_{GReX}$ and its standard error estimated by block jackknife (Quenouille, 1956), IGREX tests the tissue-specific null hypothesis that *H*_0_ : *PVE*_*GReX*_ = 0 by using a simple z-test. While IGREX is presented based on individual level data, IGREX is also applicable for GWAS summary statistics using the same model defined above. In the original study, IGREX used the GTEx project as expression mapping study and GWAS data in both individual-level and summary statistics. IGREX identified several trait-relevant tissue types. For example, significant GReX components were observed in liver for both high-density lipoprotein and low-density lipoprotein, in brain-amygdala for bipolar disorder, in brain-spinal cord (cervical c-1) for coronary artery disease, and in spleen for height.

### RhoGE

RhoGE (Mancuso et al., 2017) fits a similar model as defined in Equations (7) and (8) as IGREX, but with three differences. First, RhoGE uses only the posterior mean estimate ***μ***_*m*_ obtained from Equation (7) and subsequently ignores the uncertainty in the estimation of **w**_*m*_. Second, RhoGE is based on LDSC, and thus estimates the variance components $\sigma_{\beta }^{2}$ effectively using MoM. Third, RhoGE does not account for the horizontal pleiotropic effects ${\tilde{\text{G}}}_{r}\gamma$. Technically, RhoGE modifies the LDSC estimation procedure to use gene level summary statistics. Specifically, the gene-level statistic $\chi_{m}^{2}$ is computed as ${\hat{\text{w}}}_{m}^{T}\phi_{m}\phi_{m}^{T}{\hat{\text{w}}}_{m}/{\hat{\text{w}}}_{m}^{T}{\text{V}}_{m}{\hat{\text{w}}}_{m}$, where ${\hat{\text{w}}}_{m}$ is obtained from the genomic best linear unbiased prediction (GBLUP) (de los Campos et al., 2013); **ϕ**_*m*_ are the *p*-vector of SNP-based Wald statistics from the GWAS study; and **V**_*m*_ is an *p* × *p* LD matrix calculated from a reference panel. Afterwards, RhoGE follows the same inference procedure as in LDSC to estimate *PVE*_*GReX*_ and tests whether *PVE*_*GReX*_ is statistically significant from zero. The resulting test statistic is served as evidence for trait-tissue relevance inference. RhoGE analyzed GWAS summary statistics for 30 complex traits and found 108 significant trait-tissue pairs across 17 traits and 33 tissues, including BMI-brain, schizophrenia-brain, and high-density lipoprotein-heart.

## Methods Based on Tissue-Specific Gene Co-Expression Network

In this section, we introduce the fourth category of methods, which currently consists of only CoCoNet (Shang et al., 2020b), for trait-tissue relevance inference. CoCoNet performs trait-tissue relevance inference using tissue- or cell type-specific gene co-expression network information obtained from bulk or single cell gene expression studies. Gene co-expression networks characterize how genes are connected with each other and are coregulated together. Gene co-expression networks have been shown to be informative for predicting gene-level association effect sizes on diseases in GWASs and are often tissue and cell type specific (Chen et al., 2011; Hou et al., 2014; Jia and Zhao, 2014; Hao et al., 2018). Genes with high network connectivity have also been shown to be enriched for heritability of GWAS traits (Kim et al., 2019). Therefore, it is important to take advantage of tissue-specific gene connection information in tissue-specific gene co-expression networks to facilitate the inference of disease tissue relevance.

### CoCoNet

CoCoNet (Shang et al., 2020b) first obtains an *M*-vector of gene-level effect sizes with the trait of interest from the GWAS, denoted as $\theta ={\left(\theta_{1},\cdots \;,\;\theta_{M}\right)}^{T}$. In the gene expression study, CoCoNet examines one tissue at a time and for the given tissue constructs an *M* by *M* gene-gene adjacency matrix **A** = (*a*_*mm^′^*_) to represent the gene co-expression network there. The *mm*′-th element of the adjacency matrix $a_{mm^{\prime }}$ is 1 if gene *m* is connected to gene *m*′ in the network and 0 otherwise. *a*_*mm*_ is set to be 0 for any 1 ≤ *m* ≤ *M* to ensure the absence of self-loops (Urry and Sollich, 2013). CoCoNet then relies on a covariance regression network model (Lan et al., 2018) to model the relationship between **A** and ***θ***

(11) $$\begin{array}{l} \begin{array}{c} \theta \sim N\left(1_{M}\mu ,\Sigma \left(\text{A}\right)\right), \end{array} \end{array}$$

where μ is the intercept and Σ(**A**) is the covariance of ***θ*** as a function of the adjacency matrix **A**. The covariance Σ(**A**) is in a general form $\Sigma \left(\text{A}\right)=\overset{L}{\underset{l=0}{\sum }}\sigma_{l}^{2}{\text{A}}^{l}$, where ${\text{A}}^{l}=\left(a_{mm^{\prime }}^{\left(l\right)}\right)$ is the *l-*th power of **A**, and *L* is the maximum number of paths considered for linking between any two genes. For any integer *l*, $a_{mm^{\prime }}^{\left(l\right)}$ is the number of *l*-paths linking from gene *m* to gene *m*′ in the co-expression network, where an *l*-path is any path of length *l*. For example, when *l* = 2, $a_{mm^{\prime }}^{\left(2\right)}=\;\overset{M}{\underset{h=1}{\sum }}a_{mh}a_{hm^{\prime }}$, where $a_{mh}a_{hm^{\prime }}$ is 1 only when there is a link connecting the three genes *m* − *h* − *m*′ and 0 otherwise. For *l* ≥ 1, CoCoNet sets $a_{mm}^{\left(l\right)}=0$. When *l* = 0, CoCoNet sets **A**^0^ = **I**. In the real data application, CoCoNet suggests choosing *L* based on Bayesian Information Criterion (BIC) according to real data analysis.

Because of the computation burden associated with the model in Equation (11), CoCoNet relies on composite likelihood for approximate inference. In particular, the composite likelihood only needs to make an assumption that each pair (θ_*m*_, $\theta_{m^{\prime }}$) follows a bivariate normal distribution, instead of making a strong assumption that the *m*-vector of θ jointly follows a multivariate Gaussian distribution. Specifically, for each pair of genes *m* and *m*′, CoCoNet considers the composite likelihood $P\left(\theta_{m},\theta_{m^{\prime }}|\mu ,\sigma_{0}^{2},\sigma_{1}^{2}\right)$ as

(12) $$\begin{array}{l} \begin{array}{c} \left(\begin{array}{c} \theta_{m}\\ \theta_{m^{\prime }} \end{array}\right)\sim BN\left(\left(\begin{array}{c} \mu \\ \mu \end{array}\right),\sum_{l=0}^{L}\sigma_{l}^{2}\left(\begin{array}{cc} a_{mm}^{\left(l\right)} & a_{mm^{\prime }}^{\left(l\right)}\\ a_{mm^{\prime }}^{\left(l\right)} & a_{m^{\prime }m^{\prime }}^{\left(l\right)} \end{array}\right)\right), \end{array} \end{array}$$

where BN represents bivariate normal distribution. CoCoNet finally constructs the log composite likelihood as

(13) $$\begin{array}{l} loglik\left(\theta \right)=\overset{M}{\underset{m=1}{\sum }}\overset{M}{\underset{m^{{\;}^{\prime }}>m}{\sum }}\log P\left(\theta_{m},\theta_{m^{\prime }}|\mu ,\sigma_{0}^{2},\cdots \;,\sigma_{L}^{2}\right). \end{array}$$

CoCoNet fits the above composite likelihood through a standard maximum likelihood inference procedure. Afterwards, CoCoNet calculates the maximum composite likelihood for each tissue and eventually ranks tissues by the corresponding log likelihoods. In the original study, the comparative results between CoCoNet and LDSC-SEG/RolyPoly in the original study suggest that tissue-specific gene co-expression network provides valuable trait-tissue relevance information, perhaps more so than the information provided by marginal tissue-specific gene expression pattern used in LDSC-SEG/RolyPoly. CoCoNet analyzed eight different disease GWASs that include four neurological disorders and four autoimmune disorders on 38 tissues obtained from GTEx, CoCoNet found that the top relevant tissues identified for neurological disorders are generally brain tissues, which are disease causing tissues. CoCoNet also found the top relevant tissues for autoimmune disorders to be intestinal tissues, which are disease-target tissues. In trait-cell type relevance identification, CoCoNet found GABAergic interneurons, oligodendrocyte precursor cells, astrocytes, and microglia are the top relevant cell types in Alzheimer's disease. CoCoNet also found both pyramidal neurons and various glia cells are selected as top relevant cell types in bipolar disorder.

## Discussion

We have presented a systematic review on existing statistical methods for trait-tissue relevance inference. Our review comes from a technical perspective and summarizes the input data types, detailed statistical model and inference algorithm, criteria for evaluating tissue/cell type relevance of a trait, as well as the main findings from these existing methods. Identifying trait-relevant tissues using these methods not only facilitates the understanding of disease etiology but also enables more powerful association analysis in future GWASs (Hao et al., 2018). For example, tissue-specific SNP annotations and their contributing weights to SNP heritability in the trait-relevant tissue can be used to construct more powerful SNP set tests in GWASs (Hao et al., 2018). In addition, the inferred trait-relevant tissues and/or cell types facilities the interpretation of TWAS analysis and improves the analysis power (Gamazon et al., 2015; Gusev et al., 2016).

Thus far, existing methods have primarily relied on *ad hoc* procedures to validate the inferred trait-tissue relevance results. For example, one would examine top trait-relevant tissues one by one and look for corresponding evidence in the literature to support such results. Manually cross checking with literature, however, requires domain knowledge and may yield biased results. Manual literature checking is also time consuming and the outcome results are not easy to quantify. To overcome the shortcomings of manual literature checking, Hao et al. (2018) provided a convenient approach to quantitatively validate trait-tissue relevance identified from real data applications in an unbiased fashion. Specifically, Hao et al. (2018) performs cross checking with previous literature quantitatively via PubMed search. The intuition behind Hao's approach is that, if a tissue is truly relevant to a given trait, then the number of previous biomedical researches would have been carried out on the tissue for the trait. Consequently, the relevance of a tissue to a trait can be measured by the number of previous publications on the trait-tissue pair. Therefore, for each trait-tissue pair, Hao et al. (2018) used the names of trait and tissue as input and counted the number of publications that contain the input values either in the abstract or in the title. For example, for the schizophrenia-CNS trait-tissue pair, they conducted the search by using “schizophrenia [Title/Abstract] AND (CNS [Title/Abstract] OR brain [Title/Abstract] OR central nervous system [Title/Abstract] OR neuron [Title/Abstract] OR glia [Title/Abstract]).” By counting the number of previous publications on the trait-tissue pair, Hao et al. (2018) provides a somewhat ground truth for quantifying and comparing the inferred trait-tissue relevance results. For example, PubMed yielded 17,720 hits for the pair of schizophrenia-CNS, which covers 63.8% of all schizophrenia-tissue search results from the previous literatures, supporting the relevance between CNS and schizophrenia. By performing PubMed search, Hao et al. (2018) shows that certain histone modification marks often provide more information than others. A follow up study using similar PubMed search approach also shows that histone modifications are more informative in inferring trait-tissue relevance than using either the marginal expression information or gene co-expression network information extracted from gene expression studies (Shang et al., 2020b).

Existing methods are primarily developed to take advantage of one particular genomic information for trait-tissue relevance inference. As we summarized in the review, some methods make use of histone modification marks (for example, sLDSC and SMART) while some other methods make use of gene expression data (for example, LDSC-SEG and RolyPoly). However, different genomic information may contain complementary information for trait-tissue relevance inference. Indeed, Finucane et al. (2018) found that one function annotation may be more preferable than another. The same study thus proposed ways to combine two annotations together either by creating a joint synthetic annotation or by combining *p-*values from analyses of the two annotations separately. A follow up method, SMART, formally models multiple genomic annotations jointly with a multivariate statistical model to improve the accuracy of trait-tissue relevance inference (Hao et al., 2018). SMART found that substantial accuracy gain can be achieved by combining multiple genomic annotations than using one annotation at a time. Besides methodology development to directly incorporate multiple annotations for trait-tissue relevance inference, methods have also been developed to combine multiple annotations into a single, more interpretable and more informative annotation. For example, GenoSkyline creates synthetic annotation based on a variety of epigenetic annotations (Lu et al., 2016). An updated version of GenoSkyline, GenoSkyline-Plus, can now incorporate both RNA-seq data and DNA methylation data in addition to epigenetic annotations to produce functional epigenetic annotations across 127 tissues and cell types (Lu et al., 2017). A similar method, FUMA, is a recently developed web-based platform that can annotate GWAS significant SNPs for functional consequences on genes, CADD scores, and chromatin states in 127 tissues and cell types (Watanabe et al., 2017). Similarly, in gene expression studies, while existing approaches use either the list of tissue-specifically expressed genes, tissue-specific gene expression levels, or tissue-specific gene co-expression pattern, combining the use of all the information together may have added benefits. Therefore, developing statistical methods to incorporate multiple genomic data types as well as multiple aspects of the same data type will likely yield more accurate tissue-trait relevance in the future. Beyond the scope of our review on trait-tissue relevance, we would add a word for GWAS. GWAS has been developed and used for nearly two decades and reported over 200,000 trait-SNP associations (GWAS catalog as of Dec 15, 2020). However, sample size is always a controversial issue. Current GWAS is toward larger and larger sample sizes in order to discover novel SNPs, however, the “overly-identified” SNPs are often lack of meaningful biological explanations. In contrast, small sample size typically cannot detect any signals. The first issue is now relatively well-studied, for example fine-mapping, gene-based test, etc. We think that the second issue is worth more investigations in the field of GWAS. In addition, factors that determine the phenotype/disease are complex and various, further questions include when and how, i.e., what, when, and how a factor/factors determines a phenotype/disease. We believe that all of the theoretical, computational and experimental work are very meaningful to explore the “truth” of how genome affects “us” and makes “us” different.

## Data Availability Statement

The original contributions presented in the study are included in the article/supplementary material, further inquiries can be directed to the corresponding author/s.

## Author Contributions

All authors listed have made a substantial, direct and intellectual contribution to the work, and approved it for publication.

## Conflict of Interest

The authors declare that the research was conducted in the absence of any commercial or financial relationships that could be construed as a potential conflict of interest.
